# Leprosy: An Overview of Pathophysiology

**DOI:** 10.1155/2012/181089

**Published:** 2012-09-04

**Authors:** Ramesh Marne Bhat, Chaitra Prakash

**Affiliations:** Department of Dermatology, Father Muller Medical College, Karnataka, Mangalore 572002, India

## Abstract

Leprosy, also known as Hansen's disease, is a chronic infectious disease caused by *Mycobacterium leprae*, a microorganism that has a predilection for the skin and nerves. The disease is clinically characterized by one or more of the three cardinal signs: hypopigmented or erythematous skin patches with definite loss of sensation, thickened peripheral nerves, and acid-fast bacilli detected on skin smears or biopsy material. *M. leprae* primarily infects Schwann cells in the peripheral nerves leading to nerve damage and the development of disabilities. Despite reduced prevalence of *M. leprae* infection in the endemic countries following implementation of multidrug therapy (MDT) program by WHO to treat leprosy, new case detection rates are still high-indicating active transmission. The susceptibility to the mycobacteria and the clinical course of the disease are attributed to the host immune response, which heralds the review of immunopathology of this complex disease.

## 1. Introduction

Leprosy, also known as Hansen's disease, is a chronic infectious disease caused by *Mycobacterium leprae*, a microorganism that has a predilection for the skin and nerves. Though nonfatal, leprosy is one of the most common causes of nontraumatic peripheral neuropathy worldwide. The disease has been known to man since time immemorial. DNA taken from the shrouded remains of a man discovered in a tomb next to the old city of Jerusalem shows him to be the earliest human proven to have suffered from leprosy. The remains were dated by radiocarbon methods to 1–50 A.D. [1]. The disease probably originated in Egypt and other Middle Eastern countries as early as 2400 BCE. An apparent lack of knowledge about its treatment facilitated its spread throughout the world. *Mycobacterium leprae*, the causative agent of leprosy, was discovered by G. H. Armauer Hansen in Norway in 1873, making it the first bacterium to be identified as causing disease in humans [2, 3]. Over the past 20 years, the WHO implementation of MDT has rendered leprosy a less prevalent infection in 90% of its endemic countries with less than one case per 10,000 population. Though, it continues to be a public health problem in countries like Brazil, Congo, Madagascar, Mozambique, Nepal, and Tanzania [4].

## 2. *Mycobacterium leprae*

*M. leprae*, an acid-fast bacillus is a major human pathogen. In addition to humans, leprosy has been observed in nine-banded armadillo and three species of primates [5]. The bacterium can also be grown in the laboratory by injection into the footpads of mice [6]. Mycobacteria are known for their notoriously slow growth. With the doubling time of 14 days, *M. leprae* has not yet been successfully cultured in vitro [7, 8]. The genome of *M. leprae* has been sequenced in totality [9]. It presents with less than 50% coding capacity with a large number of pseudogenes. The remaining *M. leprae* genes help to define the minimal gene set necessary for in vivo survival of this mycobacterial pathogen as well as genes potentially required for infection and pathogenesis seen in leprosy.

*M. lepromatosis* is a newly identified mycobacterium which is described to cause disseminated leprosy whose significance is still not clearly understood [10, 11].

## 3. Genetic Determinants of Host Response

Human genetic factors influence the acquisition of leprosy and the clinical course of disease [12]. Single-nucleotide polymorphism (SNP) association studies showed a low lymphotoxin-*α* (LTA)-producing allele as a major genetic risk factor for early onset leprosy [13]. Other SNPs to be associated with disease and/or the development of reactions in several genes, such as vitamin D receptor (VDR), TNF-*α*, IL-10, IFN-*γ*, HLA genes, and TLR1 are also suggested [14–17]. Linkage studies have identified polymorphic risk factors in the promoter region shared by two genes: PARK2, coding for an E3-ubiquitin ligase designated Parkin, and PACRG [18]. A study also suggests that NOD2 genetic variants are associated with susceptibility to leprosy and the development of reactions (type I and type II) [19].

## 4. Transmission

Two exit routes of *M. leprae* from the human body often described are the skin and the nasal mucosa. Lepromatous cases show large numbers of organisms deep in the dermis, but whether they reach the skin surface in sufficient numbers is doubtful [20]. Although there are reports of acid-fast bacilli being found in the desquamating epithelium of the skin, there are reports that no acid-fast bacilli were found in the epidermis, even after examining a very large number of specimens from patients and contacts [21]. However, fairly large numbers of *M. leprae* were found in the superficial keratin layer of the skin of lepromatous leprosy patients, suggesting that the organism could exit along with the sebaceous secretions [22]. The quantity of bacilli from nasal mucosal lesions in lepromatous leprosy ranges from 10,000 to 10,000,000 [23]. Majority of lepromatous patients show leprosy bacilli in their nasal secretions as collected through blowing the nose [24]. Nasal secretions from lepromatous patients could yield as much as 10 million viable organisms per day [25]. 

The entry route of *M. leprae* into the human body is also not definitively known. The skin and the upper respiratory tract are most likely; however, recent research increasingly favours the respiratory route [26, 27]. 

## 5. Incubation Period

Measuring the incubation period in leprosy is difficult because of the lack of adequate immunological tools and slow onset of the disease. The minimum incubation period reported is as short as a few weeks and this is based on the very occasional occurrence of leprosy among young infants [28]. The maximum incubation period reported is as long as 30 years, or over, as observed among war veterans known to have been exposed for short periods in endemic areas but otherwise living in nonendemic areas. It is generally agreed that the average incubation period is between three and ten years [29].

## 6. Risk Factors

Those living in endemic areas with poor conditions such as inadequate bedding, contaminated water, and insufficient diet, or other diseases that compromise immune function are at highest risk for acquiring *M. leprae* infection. There has been concern that coinfection with HIV might exacerbate the pathogenesis of leprosy lesions and/or lead to increased susceptibility to leprosy as it is seen with tuberculosis. However, HIV infection has not been reported to increase susceptibility to leprosy, impact on immune response to *M. leprae*, or to have a significant effect on the pathogenesis of neural or skin lesions to date [30, 31]. On the contrary, initiation of antiretroviral treatment has been reported to be associated with activation of subclinical *M. leprae* infection and exacerbation of existing leprosy lesions (type I reaction) likely as part of immune reconstitution inflammatory syndrome [32–34].

## 7. Interaction of *M. leprae* with Schwann Cells and Macrophages

Schwann cells (SCs) are a major target for infection by *M. leprae* leading to injury of the nerve, demyelination, and consequent disability. Binding of *M. leprae* to SCs induces demyelination and loss of axonal conductance [35]. It has been shown that *M. leprae* can invade SCs by a specific laminin-binding protein of 21 kDa in addition to PGL-1 [36, 37]. PGL-1, a major unique glycoconjugate on the *M. leprae* surface, binds laminin-2, which explains the predilection of the bacterium for peripheral nerves [37]. The identification of the *M. leprae*-targeted SC receptor, dystroglycan (DG), suggests a role for this molecule in early nerve degeneration [38]. *Mycobacterium leprae*-induced demyelination is a result of direct bacterial ligation to neuregulin receptor, ErbB2 and Erk1/2 activation, and subsequent MAP kinase signaling and proliferation [39].

Macrophages are one of the most abundant host cells to come in contact with mycobacteria. Phagocytosis of *M. leprae* by monocyte-derived macrophages can be mediated by complement receptors CR1 (CD35), CR3 (CD11b/CD18), and CR4 (CD11c/CD18) and is regulated by protein kinase [40, 41]. Nonresponsiveness towards *M. leprae* seems to correlate with a Th2 cytokine profile.

## 8. Disease Classification

Leprosy is classified within two poles of the disease with transition between the clinical forms [42]. Clinical, histopathological, and immunological criteria identify five forms of leprosy: tuberculoid polar leprosy (TT), borderline tuberculoid (BT), midborderline (BB), borderline lepromatous (BL), and lepromatous polar leprosy (LL). Patients were divided into two groups for therapeutic purposes: paucibacillary (TT, BT) and multibacillary (midborderline (BB), BL, LL) [43]. It was recommended later that the classification is to be based on the number of skin lesions, less than or equal to five for paucibacillary (PB) and greater than five for the multibacillary (MB) form.

## 9. Clinical Features (Table 1)

### 9.1. Indeterminate Leprosy

Indeterminate (I) is a prelude to the determinate forms of leprosy [44, 45]. It is characterized by an ill-defined, bizarre hypopigmented macule(s) with a smooth or scaly surface. The sensations over the macule may or may not be impaired. The nerve proximal to the patch may or may not be thickened.

### 9.2. Polyneuritic Leprosy

Manifesting with only neural signs without any evidence of skin lesions, polyneuritic leprosy mostly well recognized in the Indian subcontinent. The affected nerves are thickened, tender, or both. Localized involvement of the nerves may form nerve abscesses [46].

### 9.3. Histoid Leprosy

Histoid leprosy is relatively uncommon, distinct clinical, and bacteriologic and histopathologic expression of multibacillary leprosy [47]. It may occur as a primary manifestation of the disease or in consequence to secondary drug resistance to dapsone following irregular and inadequate monotherapy. It manifests as numerous cutaneous nodules and plaques primarily over the back, buttocks, face, and bony prominences.

## 10. Histopathological Reactions

Histopathologically, skin lesions from tuberculoid patients are characterized by inflammatory infiltrate containing well-formed granulomas with differentiated macrophages, epithelioid and giant cells, and a predominance of CD4^+^ T cells at the lesion site, with low or absent bacteria. Patients show a vigorous-specific immune response to *M. leprae* with a Th1 profile, IFN-*γ* production, and a positive skin test (lepromin or Mitsuda reaction). 

Lepromatous patients present with several skin lesions with a preponderance of CD8^+^ T cells in situ, absence of granuloma formation, high bacterial load, and a flattened epidermis [48]. The number of bacilli from a newly diagnosed lepromatous patient can reach 10^12^ bacteria per gram of tissue. Patients with LL leprosy have a CD4 : CD8 ratio of approximately 1 : 2 with a predominant Th2 type response and high titers of anti-*M. leprae* antibodies. Cell-mediated immunity against *M. leprae* is either modest or absent, characterized by negative skin test and diminished lymphocyte proliferation [49, 50]. 

## 11. Leprosy Reactions

Leprosy reactions are the acute episodes of clinical inflammation occurring during the chronic course of disease. They pose a challenging problem because they increase morbidity due to nerve damage even after the completion of treatment. They are classified as type I (reversal reaction; RR) or type II (erythema nodosum leprosum; ENL) reactions. Type I reaction occurs in borderline patients (BT, midborderline and BL) whereas ENL only occurs in BL and LL forms. Reactions are interpreted as a shift in patients' immunologic status. Chemotherapy, pregnancy, concurrent infections, and emotional and physical stress have been identified as predisposing conditions to reactions [51]. Both types of reactions have been found to cause neuritis, representing the primary cause of irreversible deformities.

Type I reaction is characterized by edema and erythema of existing skin lesions, the formation of new skin lesions, neuritis, additional sensory and motor loss, and edema of the hands, feet, and face, but systemic symptoms are uncommon. The presence of an inflammatory infiltrate with a predominance of CD4^+^ T cells, differentiated macrophages and thickened epidermis have been observed in RR. Type II reaction is characterized by the appearance of tender, erythematous, subcutaneous nodules located on apparently normal skin, and is frequently accompanied by systemic symptoms, such as fever, malaise, enlarged lymph nodes, anorexia, weight loss, arthralgia, and edema. Additional organs including the testes, joints, eyes, and nerves may also be affected. There may be significant leukocytosis that typically recedes after the reactional state. Presence of high levels of proinflammatory cytokines such as TNF-*α*, IL-6, and IL-1*β* in the sera of ENL patients suggests that these pleiotropic inflammatory cytokines may be at least partially responsible for the clinical manifestations of a type II reaction [52, 53]. 

## 12. Immunology of Leprosy Reactions

Type I reaction is a naturally occurring delayed-type hypersensitivity response to *M. leprae*. Clinically, it is characterized by “upgrading” of the clinical picture towards the tuberculoid pole, including a reduction in bacillary load. Immunologically, it is characterized by the development of strong skin test reactivity as well as lymphocyte responsiveness and a predominant Th1 response [54, 55]. RR episodes have been associated with the infiltration of IFN-*γ* and TNF-secreting CD4^+^ lymphocytes in skin lesions and nerves, resulting in edema and painful inflammation [56, 57]. Immunologic markers like CXCL10 are described as a potential tool for discriminating RR [58]. A significant increase in FoxP3 staining was observed in RR patients compared with ENL and patients with nonreactional leprosy, implying a role for regulatory T cells in RR [59].

Pathogenesis of type II reaction is thought to be related to the deposition of immune complexes [60]. Increased levels of TNF-*α*, IL-1*β*, IFN-*γ*, and other cytokines in type II reactions are observed [61–63]. In addition, C-reactive protein, amyloid A protein, and *α*-1 antitrypsin have also been reported to be elevated in ENL patients' sera [64]. A massive infiltrate of polymorphonuclear cells (PMN) in the lesions is only observed during ENL and some patients present with high numbers of neutrophils in the blood as well. Neutrophils may contribute to the bulk of TNF production that is associated with tissue damage in leprosy. More recently, microarray analysis demonstrated that the mechanism of neutrophil recruitment in ENL involves the enhanced expression of E-selectin and IL-1*β*, likely leading to neutrophil adhesion to endothelial cells; again, an effect of thalidomide on PMN function was observed since this drug inhibited the neutrophil recruitment pathway [65]. Altogether, the data highlight some of the possible mechanisms for thalidomide's efficacy in treating type II reaction. TNF-*α* may augment the immune response towards the elimination of the pathogen and/or mediate the pathologic manifestations of the disease. TNF-*α* can be induced following stimulation of cells with total, or components of *M. leprae*, namely, lipoarabinomannan (the mycobacteria “lipopolysaccharide-” like component) a potent TNF inducer [66]. In addition, mycolyl-arabinogalactan-peptidoglycan complex of *Mycobacterium* species, the protein-peptidoglycan complex, and muramyl dipeptide all elicit significant TNF-*α* release [66].

## Figures and Tables

**Table 1:** Clinical features of leprosy.

Characteristics	Tuberculoid	Borderline tuberculoid	Midborderline	Borderline lepromatous	Lepromatous leprosy
Number of lesions	Single or upto 3	A Few (up to 10)	Several (10–30)	Numerous, asymmetrical (>30)	Innumerable, symmetrical
Size	Variable, usually large	Variable, some are large	Variable	Small, some can be large	Small
Surface changes	Hypopigmented	Dry, scaly, look bright, and infiltrated	Dull or slightly shiny	Shiny	Shiny
Sensations	Absent	Markedly diminished	Moderately diminished	Slightly diminished	Minimally diminished
Hair growth	Nil	Markedly diminished	Moderately diminished	Slightly diminished	Not affected initially
Skin smear	Negative	Negative or 1+	1–3+	3–5+	Plenty, including globi (6+)
Lepromin test	Strongly positive	Weakly positive	Negative	Negative	Negative
